# Vertebral fracture due to *Actinobacillus pleuropneumoniae* osteomyelitis in a weaner

**DOI:** 10.1186/s12917-020-02656-1

**Published:** 2020-11-11

**Authors:** Felix Giebels, Urs Geissbühler, Anna Oevermann, Alexander Grahofer, Philipp Olias, Peter Kuhnert, Arianna Maiolini, Veronika Maria Stein

**Affiliations:** 1 Division of Clinical Neurology, Department of Clinical Veterinary Medicine, Vetsuisse Faculty, University of Bern, Länggassstrasse 128, 3012 Bern, Switzerland; 2 Division of Clinical Radiology, Department of Clinical Veterinary Medicine, Vetsuisse-Faculty, University of Bern, Bern, Switzerland; 3 Division of Neurological Sciences, Department of Clinical Research and Veterinary Public Health, Vetsuisse-Faculty, University of Bern, Bern, Switzerland; 4 Clinic for Swine, Department of Clinical Veterinary Medicine, Vetsuisse Faculty, University of Bern, Bern, Switzerland; 5 Institute of Animal Pathology, Vetsuisse Faculty, University of Bern, Bern, Switzerland; 6 Institute of Veterinary Bacteriology, Vetsuisse Faculty, University of Bern, Bern, Switzerland

**Keywords:** Diskospondylitis, Abscess, Porcine, DNA sequence analysis

## Abstract

**Background:**

Osteomyelitis is relatively frequent in young pigs and a few bacterial species have been postulated to be potential causative agents. Although *Actinobacillus (A.) pleuropneumoniae* has been sporadically described to cause osteomyelitis, typically, actinobacillosis is characterized by respiratory symptoms. Nevertheless, subclinical infections are a challenging problem in pig herds. To the authors’ knowledge, this is the first case description that reports clinical, diagnostic imaging, pathological and histopathological findings of vertebral osteomyelitis in a pig and first describes *A. pleuropneumoniae* as the causative agent identified by advanced molecular methods.

**Case presentation:**

An eight-week-old female weaner was presented with a non-ambulatory tetraparesis. The neurological signs were consistent with a lesion in the C6-T2 spinal cord segments. Imaging studies revealed a collapse of the seventh cervical vertebral body (C7) with a well demarcated extradural space-occupying mass ventrally within the vertebral canal severely compressing the spinal cord. Post-mortem examination identified an abscess and osteomyelitis of C7 and associated meningitis and neuritis with subsequent pathological fracture of C7 and compression of the spinal cord. In the microbiological analysis, *A. pleuropneumoniae* was identified using PCR and DNA sequence analysis.

**Conclusions:**

*A. pleuropneumoniae* can be responsible for chronic vertebral abscess formation with subsequent pathological fracture and spinal cord compression in pigs.

## Background

Vertebral osteomyelitis is a well-known condition in food animals and has been documented in various species [1–4]. Nevertheless, to the authors’ knowledge no case report covers clinical, diagnostic imaging and pathological findings on this disease in pigs [1, 2, 5, 6].

In large animals, suppurative inflammation associated with the vertebral column is interchangeably used with a wide range of terms, i. e. *vertebral abscess* [1, 7, 8], *vertebral body abscess* [4], *spinal abscess* [9, 10], *epidural abscess* [4], *vertebral osteomyelitis* [2, 3, 9, 11], *vertebral suppurative osteomyelitis* [12], and *spinal abscess and cord compression syndrome* [13]. In swine, different compartments of the vertebral column can be affected, accordingly the inflammation is classified as *intravertebral* (i. e. osteomyelitis of the vertebral body), *paravertebral* (i. e. spondylarthritis) or *intradiscal* (i. e. diskospondylitis) [5]. In vertebral osteomyelitis in swine, main entry sites for infectious agents are the umbilical vein, bite wounds and infections after tail docking [4]. Nevertheless, it is often difficult to define the exact site of entry of the infectious agents [5, 13], since the primary infection may have resolved when the clinical signs of osteomyelitis become evident [4]. Although the etiology is most often multibacterial [8], in some cases no bacterial agent could be detected [7]. Bacterial species that have been identified in porcine osteomyelitis include *Staphylococcus aureus*, *Erysipelothrix rhusiopathiae*, *hemolytic Streptococcus*, *Pseudomonas spp.*, *Escherichia coli*, *Trueperella* (*Arcanobacterium) pyogenes, Mannheimia (Pasteurella) haemolytica* and *Actinobacillus (A.) pleuropneumoniae* [1–3, 5, 6, 14–16]. Actinobacillosis is usually characterized by pleuropneumonia [17], nevertheless, osteomyelitis and arthritis associated with *A. pleuropneumoniae* has been rarely documented [14]. Additionally, given the potential challenge to manage actinobacillosis in pig herds this case report contributes to the knowledge of atypical features of actinobacillosis.

## Case presentation

### Clinical history

An 8-week-old female weaned domestic pig with a body weight of 20 kg at acquisition was presented to the Division of Clinical Neurology, Vetsuisse Faculty of Bern, Switzerland, due to a non-ambulatory tetraparesis. Unfortunately, little was known about the history other than the weaner was found in lateral recumbency in the box on the day before presentation without prior clinical signs being noted. The pig was weaned after 4 weeks and was held in a group of 25 weaners. It was the only weaner affected within the herd.

### Clinical findings and investigation

Vital parameters revealed a rectal temperature in the lower reference range (39 °C; reference: 39.3 °C ± 0.30 °C), a moderate tachycardia (169 beats/min; reference: 90–100 beats/min), and a moderate tachypnea (60 breaths/minute; reference: 25–40/min) [18]. Other clinical findings were a stripe-formed bleeding in the subcutis of 20 cm in length and approximately 1 cm in width from the left elbow to the middle of the left scapula. Other skin lesions were detected dorsal to the left eye and in the dorsal midline of the cervical area. The tail was intact. The skin turgor was mildly reduced and the BCS was 3/5 (normal) [18].

**Fig. 1:**
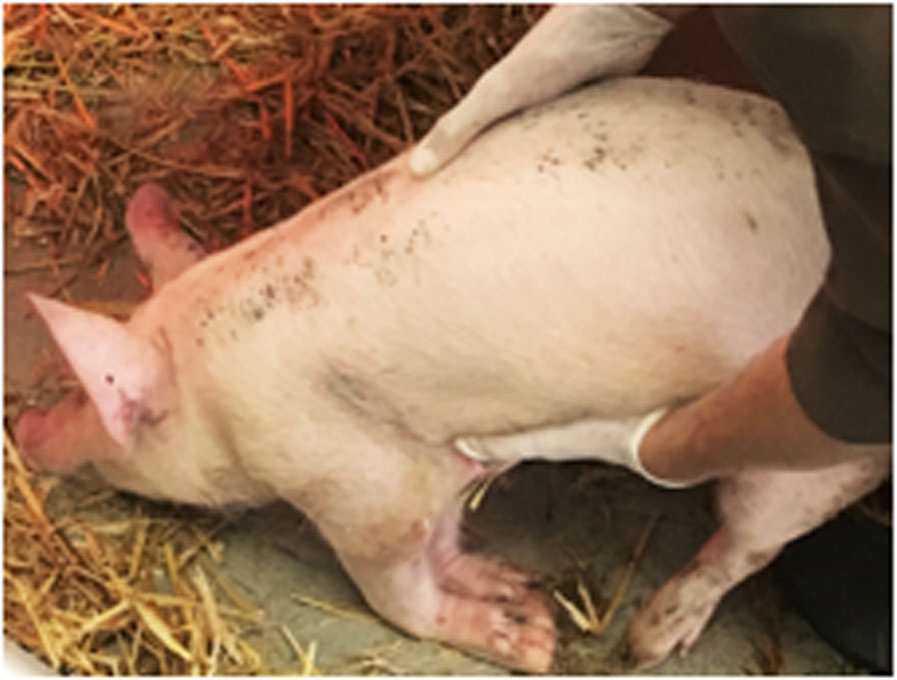
Neurological examination of the 8-week-old weaner. Assessment of the proprioception revealed severe deficits in all limbs. Note the loss of proprioception in the thoracic limbs shown here

The weaner was presented in lateral recumbency with a non-ambulatory tetraparesis. As the handling of the weaner caused significant stress in the animal, the neurological examination was abbreviated. The swine revealed a normal mental state and very stressed behavior. Proprioception seemed absent in all four limbs. Cranial nerve function was normal. The extensor tone was reduced in both thoracic and increased in both pelvic limbs (Fig. 1) and the spinal reflexes were reduced in both thoracic and normal in both pelvic limbs. The weaner seemed to be severely hyperesthetic when the caudal cervical vertebral column was palpated.

The neurological signs were consistent with a lesion localized at C6-T2 spinal cord segments [4]. Differential diagnoses comprised traumatic (i. e. vertebral fracture), inflammatory (i. e. diskospondylitis, vertebral osteomyelitis, meningomyelitis), degenerative (i. e. intervertebral disk extrusion) and vascular (i. e. spinal hematoma) etiologies.

The complete blood count showed a mild leukocytosis (22.75 × 10^9^/L; reference: 7.9–18.5 × 10^9^/L) without left-shift, a slight lymphopenia (4.78 × 10^9^/L; reference: 4.9–12.1 × 10^9^/L) and monocytosis (1.48 × 10^9^/L; reference: 0–1.37 × 10^9^/L). Blood chemistry revealed a mild hypocalcemia (2.17 mmol/L; reference: 2.32–2.92 mmol/L) and a moderately increased creatinine kinase concentration (4048 IU; reference: 0–2678 IU).

All imaging procedures were performed under general anesthesia. An intramuscular injection of medetomidine 0.08 mg/kg and ketamine 10 mg/kg was performed, reaching a sufficient sedation within 10 min which allowed to intubate the trachea. After intubation, the animal received isoflurane, which was administered in 100% oxygen.

**Fig. 2:**
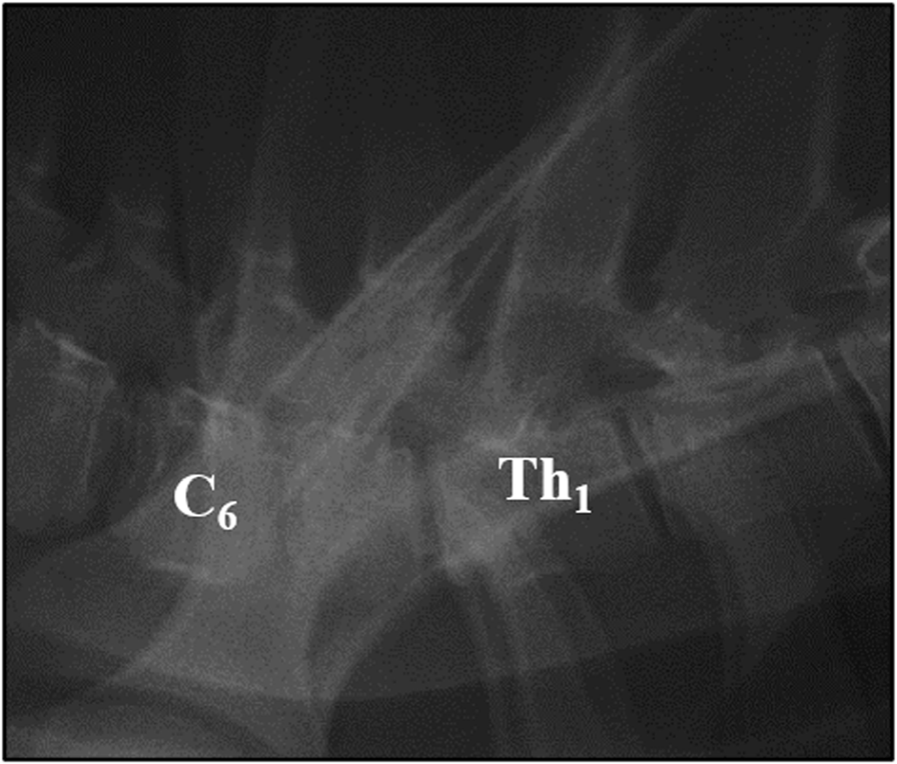
Laterolateral radiograph of the caudal cervical and cranial thoracic vertebral column of the 8-week-old weaner revealing narrowing of the C6/7 intervertebral disk space and shortening of the seventh cervical vertebral body. Note the irregular ventral margins of the seventh cervical vertebral body. C6 = sixth cervical vertebra, Th1 = first thoracic vertebra

The laterolateral radiograph of the cervical vertebral column showed shortening and irregular ventral margins of the seventh cervical vertebral body (C7) and narrowing of the C6/7 intervertebral disk space (Fig. 2). There was a regional dorsal narrowing of the trachea ventral to C6/7 by mild homogenous soft tissue thickening.

**Fig. 3:**
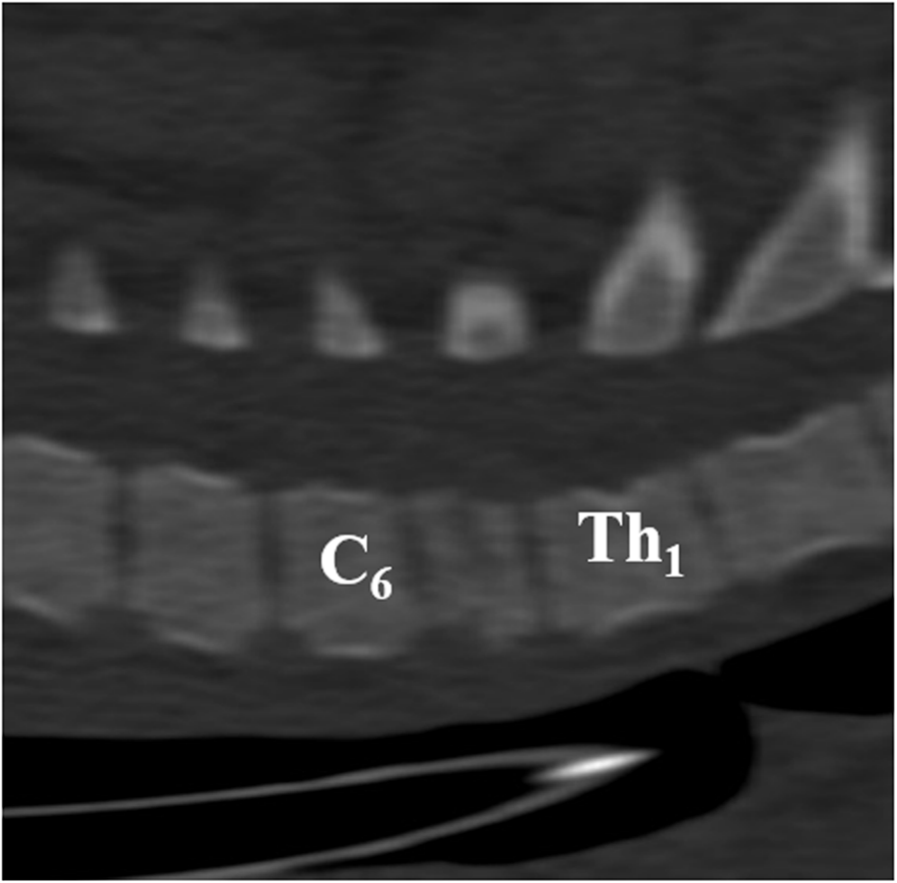
Sagittal CT reconstruction (bone window) demonstrating step formation within and shortening of the seventh cervical vertebral body of the 8-week-old weaner. C6 = sixth cervical vertebra, Th1 = first thoracic vertebra

Computed tomography (Brilliance, 16 Slice, Philips; 120 kV, 190 mAs, 2 mm slice thickness, 1 mm slice gap) of the cervical vertebral column (Fig. 3) confirmed the radiographical findings. Additionally, the vertebral body of C7 was centrally inhomogeneous and showed a mild step formation at the dorsal and ventral contour and a narrow, irregular, hypodense, dorsoventrally oriented area was present. An involvement of the vertebral canal was not identified.

**Fig. 4:**
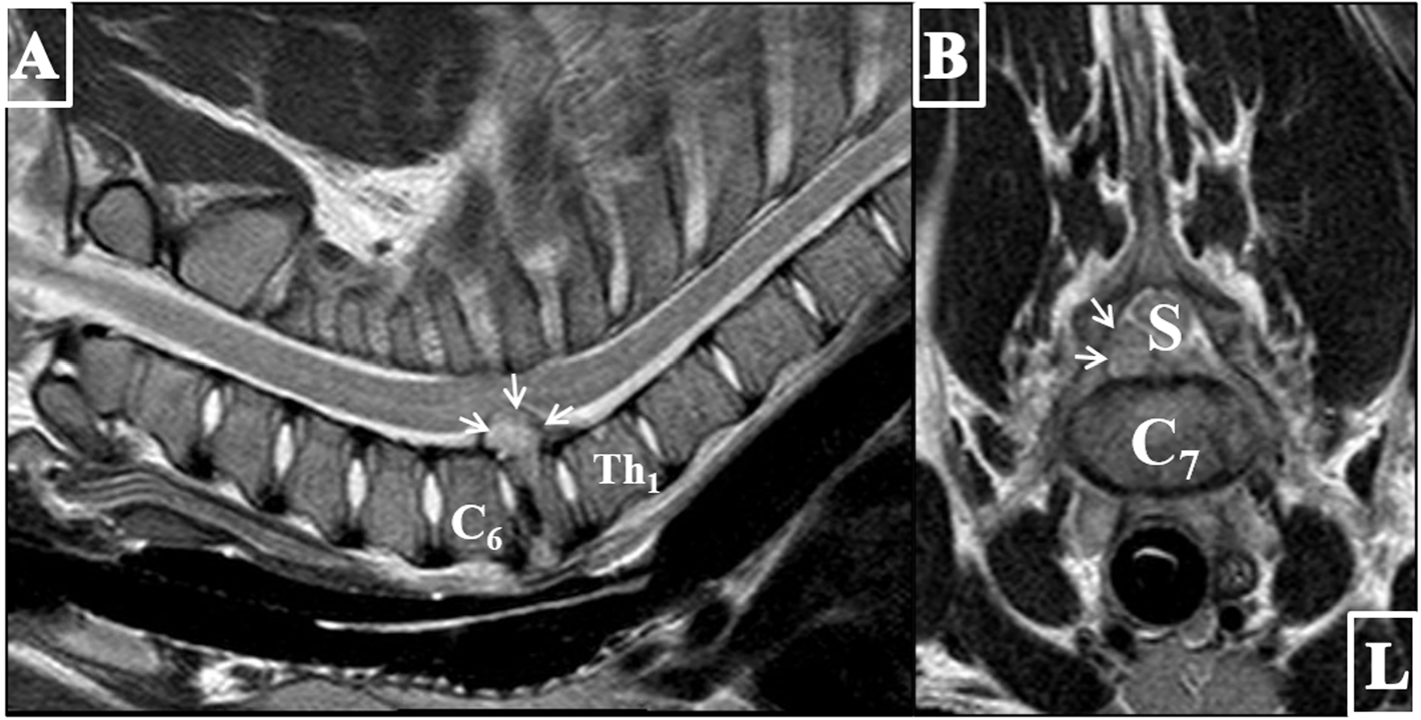
T2-weighted sagittal (**a**) and transverse (**b**) MR images of the cervical region of the 8-week-old weaner showing a collapse of the cranial aspect of the seventh vertebral body. Note the C6/7 nucleus pulposus volume reduction of approximately 50%. The C7 vertebral body reveals a heterogeneous signal intensity and an irregular, ill-defined cranial end plate. Dorsally, a well-defined, 20 mm long extradural, heterogeneous mushroom-shaped hyperintense structure (arrows) extruding into the vertebral canal in the ventral right-sided aspect of the spinal canal resulting in a compression and left dorsolateral dislocation of the spinal cord (S). The material occupied up to 75% of the vertebral canal. L = left, C6 = sixth cervical vertebra, Th1 = first thoracic vertebra

For further evaluation of the soft tissues within the vertebral canal and the paravertebral soft tissues, pre- and post-contrast (Gadolinium; Clariscan™) MRI was performed during the same anesthesia. T2-weighted sagittal and transversal, pre- and postcontrast T1-weighted dorsal and transversal, T2-weighted fat suppressed (Spectral Presaturation with Inversion Recovery (SPIR)) dorsal and T2*-weighted transversal echo gradient sequences were performed using a 1.0-Tesla open permanent magnet (Philips HFO Panorama, Philips Medical Systems, PC Best, Netherlands). MRI revealed C6/7 nucleus pulposus volume reduction of approximately 50%. The C7 vertebral body showed a heterogeneous low signal intensity in all sequences and an irregular, ill-defined cranial end plate. The vertebral body was isointense to the surrounding musculature in T1-weighted sequence with heterogeneous contrast enhancement. At the level of C7/Th1, a 20 mm long extradural, well-demarcated, heterogeneous mushroom-shaped T2/T2*/SPIR hyper- and T1 isointense, space-occupying lesion was visible in the ventral right-sided aspect of the spinal canal severely dislocating and compressing the spinal cord to the left dorsolateral side (Fig. 4a and b). The material occupied up to 75% of the vertebral canal and showed heterogenous contrast uptake. The left supra- and infraspinatus muscles were showing a stripy, ill-defined delineated hyperintensity in the T2-weighted sequence with contrast enhancement.

These imaging findings were compatible with osteomyelitis, pathological fracture and dorsal and ventral extrusion of inflammatory material (abscess) of the C7 vertebral body. Less probable differentials were primary fracture or vertebral body neoplasia.

After diagnostic imaging, the weaner was euthanized with pentobarbital (Euthasol®) during anesthesia and cerebrospinal fluid was taken atlantooccipitally immediately post-mortem. Analysis of the cerebrospinal fluid revealed a cell count of 103 cells/μl (62% neutrophils, 29% monocytes, 9% lymphocytes) with increased protein concentration (semiquantitative analysis) and negative Pandy reaction. The neutrophilic pleocytosis with increased protein pointed to an inflammatory process [4]. Main differential diagnoses for neutrophilic pleocytosis included suppurative meningitis, spinal trauma, myelomalacia and/or hemorrhage [19].

A CT-guided bone biopsy of C7 vertebral body was performed immediately post-mortem. The microscopic analysis revealed a low cellularity with a granular background containing moderate amounts of debris, smeared cell nuclei, scattered lipid vacuoles and rare pieces of striated myofibers. Intact nucleated cells represented a mixed inflammatory population, consisting predominantly of neutrophils, lower numbers of eosinophils, occasional lymphocytes and macrophages and rarely mast cells. No infectious organisms were found in Gram-stained tissue slides. Morphological diagnosis was a chronic abscess of the C7 vertebral body with a pathological fracture and compression of the spinal cord with associated meningitis and neuritis of the right-sided 6th cervical spinal nerve.

At necropsy, a dorsoventral fracture of the C7 vertebral body was present. The fracture segments were irregular and an accumulation of a moderate amount of pus extending into the spinal canal was detected adjacent to the vertebral fracture. At this location, the spinal cord was severely compressed over a length of 1.5 cm with a reddish discoloration and the dura was adhered lateroventrally to a macroscopically greyish, firm structure. The other organs were macroscopically unremarkable.

**Fig. 5:**
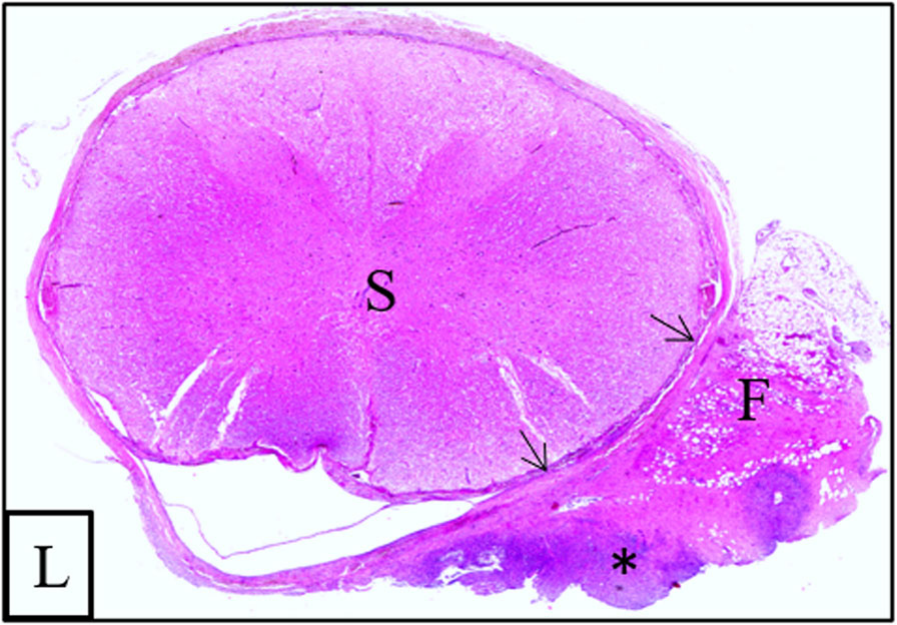
Transverse section of the spinal cord (S) at the level of the seventh cervical vertebra of the 8-week-old weaner. Histopathological examination showed a severe, focal extradural inflammation in the epidural tissue with adhesion to the dura (arrows). The inflammation infiltrated the epidural fat tissue (F) and was centrally compound of mainly degenerated neutrophilic granulocytes (asterisk) surrounded by numerous macrophages and fibrous tissue. L = left

Histologically, a severe focal-extensive inflammatory process consisting of numerous degenerated neutrophils surrounded by a high number of macrophages within proliferated fibrous tissue (abscess, Fig. 5) was present in the epidural tissue of the cervical spinal cord and attached to the dura. This inflammatory process involved emerging spinal nerves and paraspinal fat tissue. Within the compressed spinal cord disseminated swollen axons were detected within the white matter. The morphological diagnosis was a chronic abscess of the C7 vertebral body with a pathological fracture and right-sided compression of the spinal cord with associated meningitis and neuritis of the 6th cervical spinal nerves.

Thin slices of the paraffin-embedded vertebral body of C7 were used for PCR and DNA sequence analysis. DNA was prepared from paraffin slices using a previously established protocol [20] followed by column purification using the High pure PCR purification kit (Roche Diagnostics, Rotkreuz, Switzerland). An approximately 360 bp PCR product was amplified with primers 16SUNI-L and 16SRNAV-S using 2 μl of purified paraffin extract and subsequently sequenced according to the protocol described by Kuhnert et al. [21]. Sequences proof-read in both directions were compared against GenBank using BLAST.

A 100% match was found with the *A. pleuropneumoniae* type strain 16S rRNA gene sequence (Acc.no. NR_115546).

## Discussion and conclusion

Herein, we present the first case report of vertebral osteomyelitis in a pig caused by *A. pleuropneumoniae*.

In pigs, neurological examinations are rarely performed since their handling is hindered as they become rapidly stressed [4, 8, 22]. Although not all tests could be performed, it was still possible to assign the clinical signs to a C6-T2 myelopathy. Based on the sudden onset of tetraparesis differential diagnoses included a traumatic, inflammatory, degenerative, or vascular etiology. Due to the presence of cervical hyperesthesia, a vascular etiology seemed less likely. Although rarely described in swine, intervertebral disk extrusion is reported in the Yucatan pig that share some anatomic similarities with chondrodystrophic dog breeds potentially predisposing them to disk degeneration [23].

Diskospondylitis and osteomyelitis were considered the most likely differential diagnoses due to the young age and the presence of typical clinical signs [2, 4, 7, 12, 16]. Although diskospondylitis, vertebral osteomyelitis and associated vertebral fractures are mainly found in the lumbar or lumbosacral area in pigs, about 30% of reported vertebral abscesses are distributed within the cervical segments [1, 2, 5, 7, 8]. Likewise, in the dog diskospondylitis predominantly affects the lumbosacral intervertebral disks [24, 25] but can occur in the disk between the seventh cervical and first thoracic vertebra [24]. It was assumed that a high range of motion and/or the height of the disk might be associated with a predisposition for diskospondylitis. Wilke et al. [26] showed a relatively high range of motion in the porcine C6/7 segment during extension/flexion and lateral bending movements hence potentially rendering this site prone to develop diskospondylitis.

There is strong disagreement in the literature concerning the focus of the vertebral column infections in swine, making very difficult to localize the initial site of bacterial infection. Although the intradiscal structures and the adjacent bone have been described to be extensively affected in many cases [5], other authors consider the damage of these structures to occur only in rare cases [8]. This disagreement is also reflected by the existing plethora of terms describing the pathological findings in diskospondylitis/osteomyelitis in swine. In the presented case the epicenter of the disease is localised within the vertebral body, making the term osteomyelitis more appropriate. However, the cause of the narrowed C6/7 intervertebral disk space and the C6/7 nucleus pulposus volume loss remain unclear. Possible explanations might be chronic nucleus pulposus dehydration or acute nucleus pulposus extrusion. Confirming imaging findings for the latter process and histopathologic description of the C6/7 intervertebral disk are lacking.

Osteomyelitis may lead to pathological fractures, vertebral collapse and compression of the spinal cord [8]. In fact, Nietfeld [22] described vertebral fractures and abscesses as the most common cause of spinal abnormality in food animals. A trauma such as being temporarily stuck in a fence was suspected in the weaner presented based on the ecchymoses in the subcutis in the area of the lesion.

It must be taken into account that in contrast to pet animals, in which signs of spinal pain and/ or neurological deficits are generally noticed early by the owner, signs in herded livestock animals are frequently detected at a later stage. Thus, the clinical, neurological and radiological signs are frequently more severe at presentation as the pathological process has further progressed (i. e. pathological fracture). Additionally, the radiographic appearance of vertebral osteomyelitis can differ between young and adult dogs but also between acute and chronic conditions [27]. Since the onset of osteomyelitis or of first (subtle) symptoms could not exactly be determined in the weaner it is difficult to estimate the stage of inflammation. However, the pathohistological findings and the presence of a pathological fracture give evidence to a chronic process.

The MRI appearance of vertebral osteomyelitis is poorly documented in veterinary literature [28]. Nevertheless, changes include a disruption of the normally hypointense cortex on T1- and T2-weighted images and abnormal signal intensity in the vertebral body with various degrees of patchy contrast enhancement in dogs [28]. Rabillard et al. [29] described a T1-weighted hyperintensity of the vertebral body and spinal cord, which is in contrast to the described isointensity in the T1-weighted sequence herein. Nevertheless, in a large cohort study on canine diskospondylitis all affected vertebral bodies revealed a low signal intensity compared to normal bone marrow [25]. These findings are supported by MRI findings in human patients suffering from pyogenic vertebral osteomyelitis [30].

In pigs, many bacterial species have been identified in vertebral osteomyelitis. Identification of the exact entry point of the bacterial invasion could be challenging in swine since the entrance might have been healed [4]. This is consistent with the described pathological findings here, in which a chronic abscess has been diagnosed but the location of bacterial entry could not be identified. The most commonly demonstrated species are *Corynebacterium pyogenes* and *hemolytic Streptococcus spp.* [4, 7, 8]. *A. pleuropneumoniae* is the cause of contagious pleuropneumonia in pigs [8, 14]. Kaige et al. [16] demonstrated pleural abscesses following *A. pleuropneumoniae* infection in two pigs leading to paralysis of the pelvic limbs. Jensen et al. [14] demonstrated *A. pleuropneumoniae* associated osteomyelitis in multiple joints via in-situ hybridization and concluded an infection following hematogenous spread. In both reports it has been reported that the animals itself [16] or the herd in which the pigs were held [14] revealed respiratory problems. Nevertheless, subclinical infections with *A. pleuropneumoniae* are a challenging problem in pig herds [17]. Interestingly, the pathological examination did not reveal any findings in the respiratory system or any other organ. One limitation of the current case report is the lack of a microbiological culture of a lesion sample due to retrospective microbial analysis of the case. Nevertheless, we were able to diagnose presence of *A. pleuropneumoniae* in the clinical material by 16S rRNA gene amplification and sequencing.

The use of ribosomal RNA gene amplification and sequencing has been applied efficaciously for microbial diagnostics for many years in our laboratory [31–37]. These include several clinical cases and the use of embedded material of a paraffin block as successfully done in the present report confirming the potential of the approach for retrospective analyses.

To the authors’ knowledge, this is the first case report demonstrating the clinical, diagnostic imaging, (histo-)pathological, and microbiological findings in the porcine species suffering from vertebral osteomyelitis and abscess formation. Our findings add diagnostic imaging information on vertebral osteomyelitis in pigs. *A. pleuropneumoniae* was identified in a vertebral abscess and could have been the etiological cause of the vertebral osteomyelitis and subsequent vertebral fractures.

*A. pleuropneumoniae* could be responsible for chronic vertebral abscess formation with subsequent pathological fracture and spinal cord compression.

## Data Availability

All data generated or analysed during this study are included in this published article.

## Abbreviations

μl: Microliters
A. pleuropneumoniae: Actinobacillus pleuropneumoniae
BCS: Body condition score
BLAST: Basic Local Alignment Search Tool
bp: Base pairs
C: Cervical
CT: Computer tomography
DNA: Deoxyribonucleic acid
IU: International units
min: Minute
mmol/L: Millimoles per liter
MRI: Magnetic resonance imaging
PCR: Polymerase chain reaction
RNA: Ribonucleic acid
rRNA: Ribosomal ribonucleic acid
SPIR: Spectral presaturation with inversion recovery
Th: Thoracic
